# Preparing for the next pandemic via transfer learning from existing diseases with hierarchical multi-modal BERT: a study on COVID-19 outcome prediction

**DOI:** 10.1038/s41598-022-13072-w

**Published:** 2022-06-24

**Authors:** Khushbu Agarwal, Sutanay Choudhury, Sindhu Tipirneni, Pritam Mukherjee, Colby Ham, Suzanne Tamang, Matthew Baker, Siyi Tang, Veysel Kocaman, Olivier Gevaert, Robert Rallo, Chandan K Reddy

**Affiliations:** 1 Pacific Northwest National Laboratory, Richland, 99354 USA; 2 Department of Computer Science, Virginia Tech, Arlington, 22203 USA; 3 Department of Medicine, Stanford Center for Biomedical Informatics Research, School of Medicine, Stanford University, Stanford, 94305 USA; 4 Department of Biomedical Data Science, Stanford University, Stanford, 94305 USA; 5 Department of Electrical Engineering, Stanford University, Stanford, 94305 USA; 6 Division of Immunology and Rheumatology, Department of Medicine, Stanford University, Stanford, 94305 USA; 7 John Snow Labs, Delaware City, 19958 USA

**Keywords:** Computer science, Health care, Predictive markers, Risk factors

## Abstract

Developing prediction models for emerging infectious diseases from relatively small numbers of cases is a critical need for improving pandemic preparedness. Using COVID-19 as an exemplar, we propose a transfer learning methodology for developing predictive models from multi-modal electronic healthcare records by leveraging information from more prevalent diseases with shared clinical characteristics. Our novel hierarchical, multi-modal model (${\textsc {TransMED}}$) integrates baseline risk factors from the natural language processing of clinical notes at admission, time-series measurements of biomarkers obtained from laboratory tests, and discrete diagnostic, procedure and drug codes. We demonstrate the alignment of ${\textsc {TransMED}}$’s predictions with well-established clinical knowledge about COVID-19 through univariate and multivariate risk factor driven sub-cohort analysis. ${\textsc {TransMED}}$’s superior performance over state-of-the-art methods shows that leveraging patient data across modalities and transferring prior knowledge from similar disorders is critical for accurate prediction of patient outcomes, and this approach may serve as an important tool in the early response to future pandemics.

## Introduction

The COVID-19 pandemic revealed salient challenges in developing systems that can accurately predict outcomes associated with an emerging infectious disease. In particular, it emphasized the need for hospitals to access risk stratification tools that could be used to proactively identify COVID-19 patients at a greater risk of undesirable outcomes^1–9^. Such capabilities are critical for institutions to prioritize resources, and bring a quantitative approach to triaging^10^ in an emergency, which subjects the human caregivers to intense psychological stress. Undertaking hard and pragmatic decisions, and accepting their consequences leads to a new crisis that is appropriately called the “hidden pandemic for healthcare workers”^11–13^. Due to the lack of historical COVID-19 cases for training supervised machine learning models, early methods for COVID-19 severity prediction focused on the analysis of a relatively small number of carefully chosen model covariates, which included demographic risk factors, prior comorbidities, symptoms on admission, and laboratory biomarkers^8,14^. These carefully chosen covariates were predominately used to train multivariate logistic regression and boosted decision tree-based approaches^1,15–17^. Electronic health records are heterogeneous data sources that include unstructured clinical notes, structured data that are coded as ICD diagnoses and CPT procedures, and numeric measurements such as body vitals along with various laboratory test results. Due to their size, richness, and wide-scale adoption, the past few years have seen major progress in developing predictive models for different subsets of such data sources, where deep learning methods have been shown to achieve state-of-the-art results for several medical outcomes such as re-admissions, mortality prediction, and length of stay^14,18–27^. However, a notable gap lies in integrating all of the multi-modal information into a single predictive model, and the challenges are amplified by the need for large amounts of training data. In this paper, we propose ${\textsc {TransMED}}$, a methodology for developing multi-modal predictive models, while addressing training data scarcity issues posed by emerging (or rare) diseases through transfer learning from diseases with shared cohort-level characteristics and similar outcomes.

To address the existing gaps in pandemic preparedness, we sought to improve on current methods to: (i) predict if a patient will be staying in the hospital, after a certain time using the patient’s multi-modal history. This provides a better understanding of the severity of a patient’s condition, and (ii) predict the likelihood of a patient requiring mechanical ventilation. Collectively, these prediction tasks capture the inherent challenges of inpatient resource planning such as those to predict which patients are most likely to experience poor outcomes over a span of next 3-7 days^10,28–31^.

**Figure 1:**
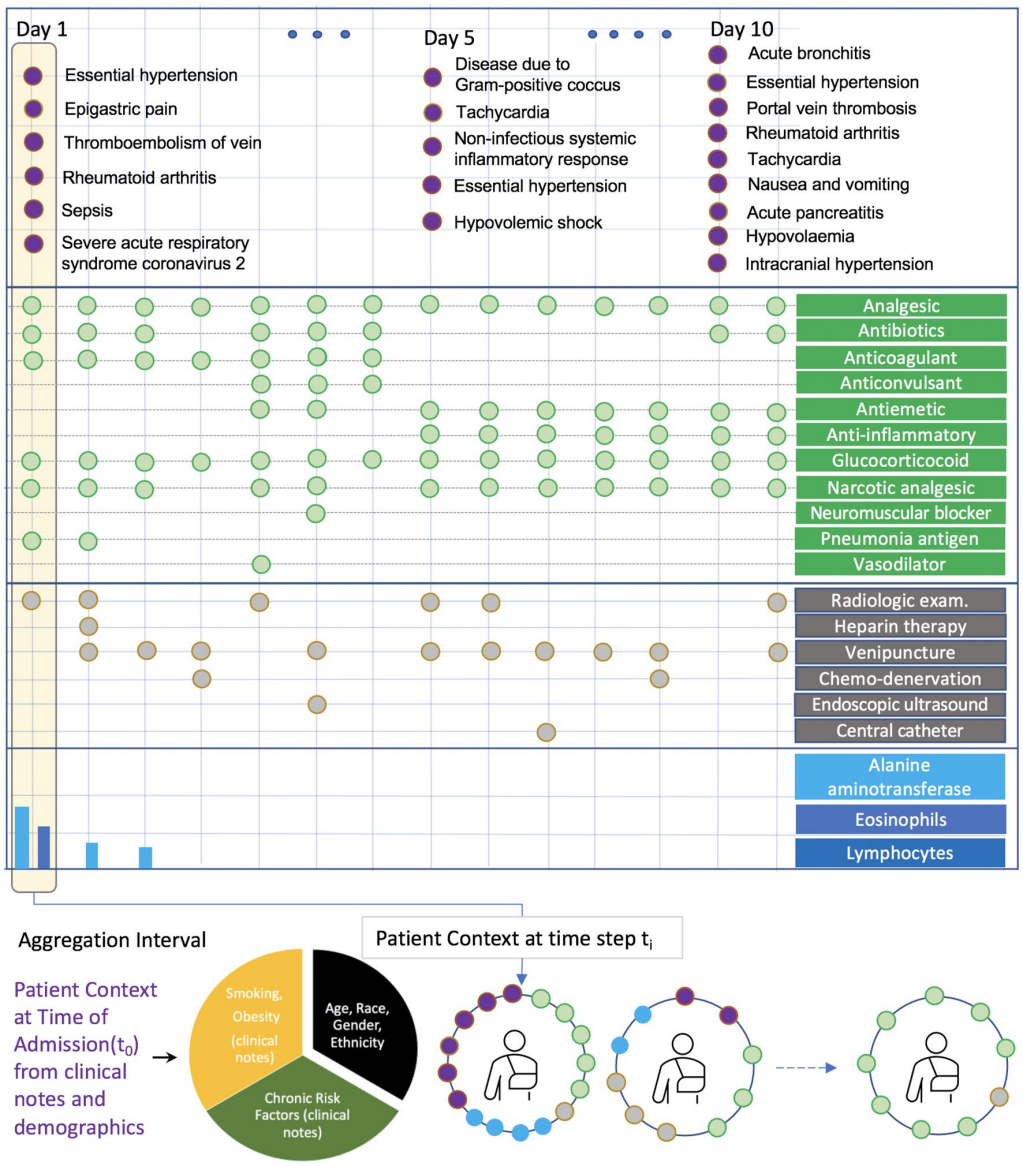
An illustration of multi-modal data sources observed over the course of a COVID-19 patient’s stay in the hospital. The colors indicate diagnosis (purple), drugs (green), procedures (gray), and numeric lab measurements (blue bars). Different data modalities are observed at varying frequency in raw patient data, with lab measurements being the most sparse across patients and across time. ${\textsc {TransMED}}$ reduces the impact of sparsity by utilizing all modalities of data in a given time interval (e.g., 24 h), creating more informed patient state snapshots in time.

Figure 1 presents a case study of a real COVID-19 patient to illustrate how different modalities offer unique information to reason about a patient’s current state and future evolution. However, making use of the information requires careful handling of sparsity across time and data sources. Diagnostic codes provide a more definitive assertion of patient’s short- and long-term medical conditions, but they do not provide continuous observation of the patient. In addition, they are not frequently observed and may miss key signals related to clinical deterioration. Observing the occurrence of key procedures and laboratory measurements provide clinical information on a patient’s immediate conditions. The drug data stream provides additional treatment information that helps characterize a patient’s disease state; compared to clinical observations such as diagnostic codes, procedures, and laboratory tests, which typically marks the onset of a problem, the duration of a medication allows us to reason about the type and severity of a particular symptom and it’s short- and long-term consequences; thereby serving as a bridge to connect other data sources that more sporadically report information.

### Our contributions

${\textsc {TransMED}}$ uses a hierarchical approach for learning cross-modal interactions across medical concepts that occur closely in time. The self-supervised methodology implemented via BERT layers^32^ is first used to learn the higher-order fine grained medical concept interactions using a Severe Respiratory Disease (SRD) cohort from Stanford Hospital with 9348 patient hospitalizations. Next, specific layers of the model are further trained for modeling temporal trajectories of COVID-19 patients using EHR data of 1701 patients. Our neural architecture is distinct from the other recent BERT-based^32,33^ prediction models in multiple dimensions. Our model integrates temporal information in multiple representations, that includes clinical notes, discrete entity-based representation of diagnostic codes, drug codes, procedure codes along with continuous valued time-series measurement of laboratory tests. To the best of our knowledge, the proposed model demonstrates the widest integration of EHR-based data sources across multiple modalities for COVID-19 severity prediction. Our experiments show that our hierarchical transfer learning based approach using Severe Respiratory Disease (SRD) cohorts leads to an average improvement of 12.9% and 10.3% in AUROC for COVID-19 patient stay and ventilation prediction. We benchmark our implementation with three models representing distinct prediction approaches and demonstrate an improvement ranging over 5.8–29.2% for AUROC and 3.6–66% in F1 score measure for ventilation prediction tasks, and accurately predicting the likelihood of short- and long-term patient stays.

We also present a new methodology to interpret and evaluate model predictions via multi-comorbidity analysis. Much of the literature on EHR models focus on characterizing the prediction performance through univariate analysis of the well understood risk factors^30,34^. However, clinical presentation of a disease and it’s severity can markedly vary depending on the constellation of symptoms, prior health conditions and risk factors. Deep learning models are adept at learning higher-order feature interactions. Therefore, evaluating model recommendations solely in terms of single factors may not explain when a prediction is driven by a hidden combination of multiple factors. We present a methodology for identifying top multi-comorbidity conditions in a data-driven fashion and evaluate their relative impact on model predictions. We believe our analysis would motivate data-driven discovery of key multi-comorbidities associated with a disease while advancing the interpretability and rigor for evaluating deep learning models for clinical use.

## Methods

We begin this section with a description of the available data sources and the cohort selection process. After that, the problem statement is described followed by a description of the model architecture.

### Data sources

Our study is based on de-identified EHR data of all patients treated at Stanford Hospital, between January 1, 2015 and March 19, 2021. This dataset was provided via STAnford Research Repository (STARR)^35^ and was used under approval by Stanford University Institutional Review Board (IRB) protocol: 50033 (Machine Learning of Electronic Medical Records for Precision Medicine). Patient informed consent was waived by Stanford University Institutional Review Board (IRB) for this protocol. All methods were carried out in accordance with relevant guidelines and regulations.

**Figure 2:**
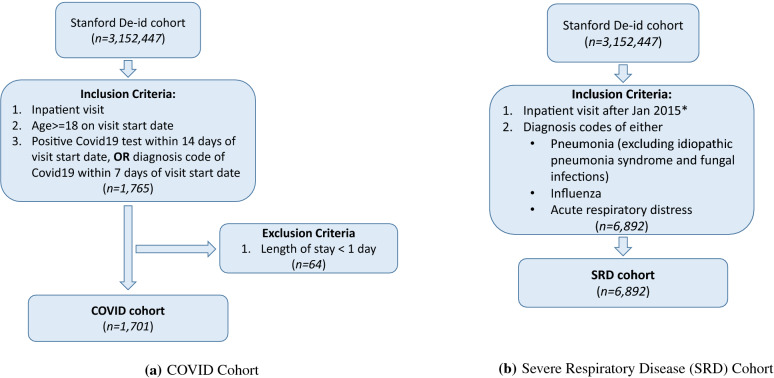
Cohort selection process for COVID-19 and Severe Respiratory Disease (SRD) patients from the Stanford Hospital. *For the SRD cohort, the start year 2015 was chosen heuristically to ensure sufficient data.

As part of the de-identification process, the actual admission dates were randomized up to 30 days. The data was retrospectively collected during the practice of care and transformed into the OMOP Common Data Model Version 5, (https://www.ohdsi.org/data-standardization/the-common-data-model/) by the STARR OMOP team. Using the STARR OMOP data, we created our cohort of COVID-19 patients (Fig. 2) based on the following inclusion criteria: (1) patients with inpatient visits after January 1, 2020, (2) patient age greater than 18 at admission, (3) patient had either a positive COVID-19 test within 14 days prior to the admission or had a diagnosis of COVID-19 within 7 days prior to the admission. Visits that were less than 1 day in length were excluded. We also created a cohort that included hospital admissions for severe respiratory disease (SRD) patients with influenza, pneumonia or ARDS, for our transfer learning approach. The ICD-9 codes were first mapped to OMOP CDM V5 concept identifiers which were then used to execute queries to retrieve the cohort data. The specific codes used for the cohort selection and mechanical ventilation are listed in the Supplementary Table 1. The cohorts were cross-referenced for similarity in observed medical codes and ventilation outcomes. Table 1 provides the summary statistics for the two cohorts, with a detailed comparison under the results section.

Our study cohorts include clinical observations from four data sources: (1) free-text patient notes at the time of admission, (2) discrete codes (we also refer to them as structured data) representing diagnosis codes, prescribed drugs, laboratory tests that were ordered, and codes for the procedures performed, (3) continuous time-series measurements that were available for a subset of the ordered laboratory tests, and (4) patient demographics (age/race/sex/ethnicity). We parsed each note into sections and used the SparkNLP library^36^ named entity recognizer (NER) for extracting medical conditions from the clinical notes (see Supplementary section on “Data Sources” for implementation details). The extractions were used to determine the presence or absence of baseline risk factors for each patient at the time of admission, including: Coronary Artery Disease (CAD), diabetes, family history, hyperlipidemia, hypertension, existing medication, obesity, and smoking. Note that COVID-19 patients were modeled de novo, using only data from COVID-19 related admission. Family history and existing medication were discarded due to insufficient coverage.

### Problem statement

Let $X_t = (C_t, M_t)$ be the patient state at time *t*, where $C_t\subseteq {\mathscr {C}}$ is a set of observed codes, $M_t\in {\mathbb {R}}^{|M|}$ is a vector of lab values, and ${\mathscr {C}}$ and |*M*| denote set of codes and lab values observed across the whole cohort respectively. Let $d\in \ \{0,1\}^{|D|}$ and $r\in \{0,1\}^{|R|}$ be multi-hot vectors denoting patient demographics and risk factors obtained at the time of admission, respectively. The clinical outcome at time *t* is denoted by $O_t$. The problem statement is as follows: Given demographics *d*, risk factors *r*, and a sequence of $T_h$ historical states $X_{t-T_h+1}, X_{t-T_h+2}, \ldots , X_t$, predict a clinical outcome of interest $T_f$ steps ahead into future, denoted by $O_{t+T_f}$. Following the tasks proposed in the introduction section, we will focus on two outcomes: patient staying at the hospital or patient requiring mechanical ventilation at time $(t+T_f)$. $T_h$ and $T_f$ are referred to as “# input time-steps” and “look-ahead”, respectively.

### Model architecture

**Figure 3:**
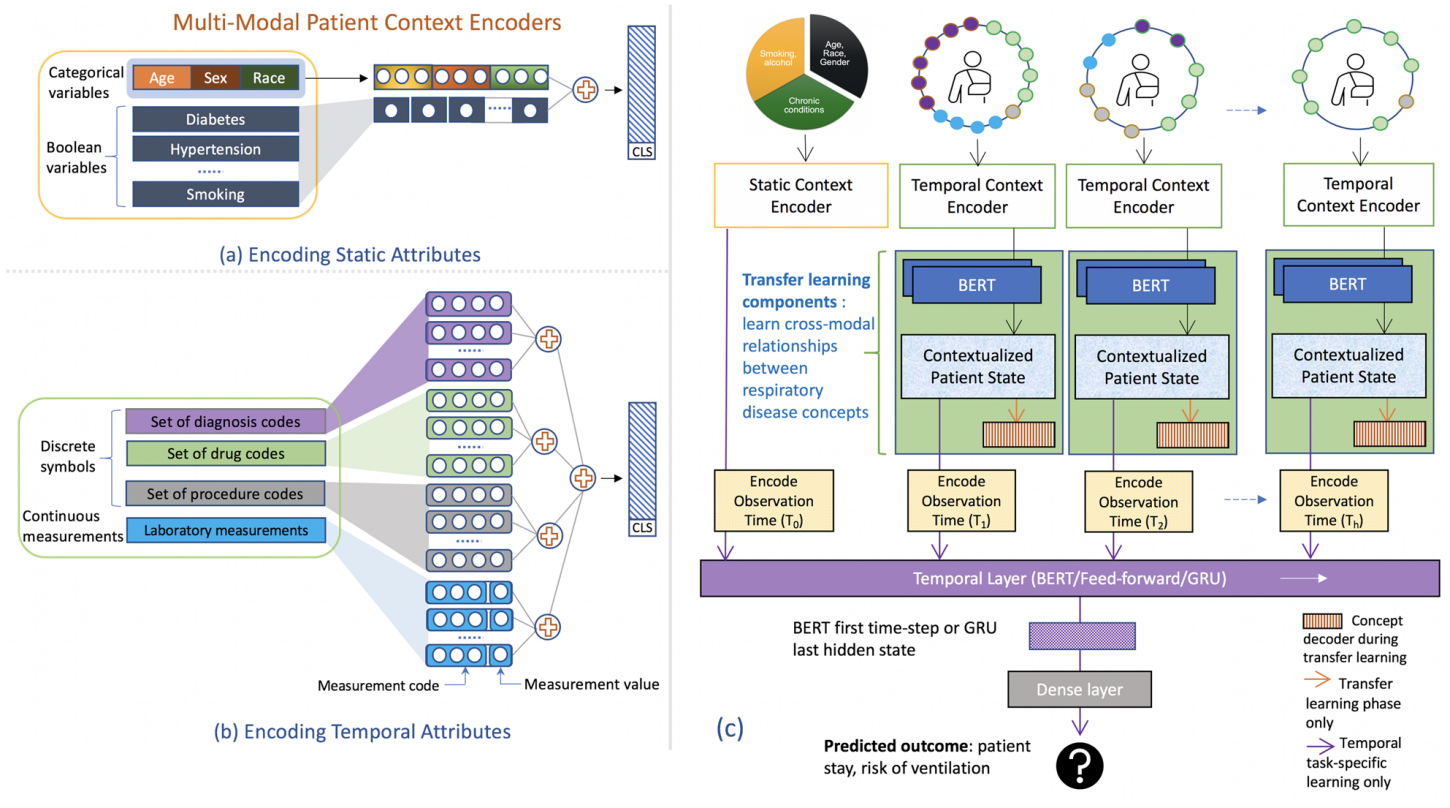
${\textsc {TransMED}}$ architecture. Patient context encoders are shown in (**a**) for static attributes and (**b**) for multi-modal temporal attributes. The proposed hierarchical transfer learning model is shown in (**c**). The transfer learning components take as input the patient’s multi-modal encoded state and produce a contextualized vector. The vectors for all time steps are combined along with static attributes to model patient’s (task-specific) evolution over time.

In this section, we present the intuition behind the key components of our model and provide details of the proposed hierarchical model architecture. The supplementary section titled “Related Work” provides a detailed overview of all prominent methods that has been applied over diverse modalities and prediction methodologies for EHR data. Figure 3 presents the data-flow through our hierarchical multi-modal model with two primary components: (1) Transfer learning driven top layers that accept patient state at a given time (subsequently referred to as “patient context”) as input and produces a contextualized representation, capturing feature interactions across all modalities of data in a single time step. (2) The bottom temporal modeling layer to model patient evolution over time. This layer takes as input, the contextualized vector representations output from top layers for each time interval augmented with positional encoding (indicating the time of observation) and produces a vector representing patient evolution over time.

The transfer learning component is implemented using BERT layers due to their proven effectiveness in learning contextual relationships between set of observations^32^. The choice for specific implementation of the temporal modeling layer is flexible, and we discuss the different options for this layer in detail below. Overall, we use the SRD cohort to train the cross-modal interaction layers during transfer learning. We train the temporal layers on the COVID-19 patient data only and keep the multi-modal transformer layers fixed from the training of SRD cohort.

#### Multi-modal patient context encoders

 We begin with describing the “patient context” data structure that captures the state of a patient over a single aggregation interval, across all modalities of patient data. We define the patient context as a collection of labeled sets that contains both static attributes (such as demographics and risk factors) and all multi-modal EHR information available during a specific interval in time.

We map numeric age values into 11 bins, based on CDC criteria (https://www.cdc.gov/coronavirus/2019-ncov/covid-data/investigations-discovery/hospitalization-death-by-age.html). Age, race, and ethnicity are represented as categorical variables, while the rest of the variables such as sex and presence of risk factors are represented as boolean variables (shown in Fig. 3a). The temporal information associated with the patient context at a given time interval can be naturally recognized as a collection of sets, where each set represents a type of information such as diagnosis codes, drugs, procedures, laboratory tests and contains the discrete identifiers of associated diagnosis codes, drugs, etc. (shown in Fig. 3b). The multi-modal patient context encoder module takes in the collection of sets as described above and returns a single vector representing the patient context. We encode each boolean and categorical variables as a one-hot vector. The last component in the patient context are laboratory measurements, which are represented as a dictionary of key-value pairs representing a test and it’s associated numeric value. We only used the laboratory measurements from the COVID-19 cohort due to unit consistency issues in the SRD data. A concatenation of all these vectors yields the final embedding, representing the patient context.

#### Learning contextual representation of patient state in a time interval

Our first component focuses on learning a contextualized representation of the patient state per time interval. This *contextualization* is important. For example, a drug may be recommended as an “if-then” measure, where it is used if the patient descends into a critical condition. If a physician were to read a chart with such information, they would understand this series of events and recognize why the drug exists on the patient record. On the contrary, if the patient was already in a condition where the drug had to be administered, it would be reflected through the presence of other diagnostic codes and lab measurements. The BERT layer in Fig. 3 accomplishes this contextualization effect, and generates a different vector embedding for each entity (such as the drug) depending on the input patient context.

#### Transfer learning

 Our unique hierarchical transfer learning exploits short-term similarity across SRD and COVID-19 cohort, while accounting for differences in their patient’s temporal trajectories. We adopt a self-supervised learning approach^37,38^ to first train the BERT-based multi-modal patient context encoder layer (green box in Fig. 3c) on short-term patient state representation. Given a patient context $C_t=(c^1_t,\ldots ,c^{|C_t|}_t)$, we generate a random mask $m_t\in \{0,1\}^{|C_t|}$ to replace a specified number of condition, drug or procedure codes with a special [*MASK*] token. and train the model to predict the missing values from the rest of the patient context using categorical cross-entropy loss.

1 $$\begin{aligned} {\hat{C}}_t = ({\hat{c}}_t^1,\ldots ,{\hat{c}}^{|C_t|}_t) \quad \text {where} \quad {\hat{c}}^i_t = {\left\{ \begin{array}{ll} [MASK], &{}\text {if }m_t[i]=1 \\ c^i_t, &{}\text {otherwise} \end{array}\right. } \end{aligned}$$

Unlike existing self-supervised methods for EHR data^20,32,33^ that mask and predict codes encoded within the complete patient trajectory, we perform contextualization within a small time step (e.g., 12–24 h) without encoding time. This facilitates learning a more fine-grained contextualization model from SRD, which is essential for transfer learning across diseases where the patient conditions may evolve at different time-scales^7,15^. The masked patient context ${\hat{C}}_t$ is then passed through a BERT module to get contextualized patient state at time *t*. $h^{C}_t$ denotes the embedding for the entire context.

2 $$\begin{aligned} h^{C}_t, h^1_t, \ldots , h_t^{|C_t|}&= BERT({\hat{c}}_t^1, \ldots , {\hat{c}}^{|C_t|_t}) \end{aligned}$$

which are passed through a linear layer to predict the masked codes.

3 $$\begin{aligned} \tilde{c_t}^i&= softmax(w^Th^i_t) \in {\mathbb {R}}^{|{\mathcal {C}}|} \quad \forall i=1,\ldots ,|C_t| \end{aligned}$$

#### Learning over time from the entire history

Finally, the temporal modeling step introduces additional layers on top of the pre-trained layers to specialize the model for specific prediction tasks. All encoded patient contexts ($h^{C}_i, i=1, \ldots , T_h$) are further augmented with a relative positional encoding ($r^{pos}_i$)^37^ to produce the inputs (denoted as $\hat{X_i}$ below) going into the temporal layer (purple box in Fig. 3c). The position encoding represents the offset in time as measured from the beginning of the patient stay, and allows us the model to reason about variable gaps in the patient data. For example, assuming we aggregate a patient’s information using a 24 h interval, and we have 3 diagnosis codes being reported at 3 PM (day 1), 9 AM (day 3), and 3 AM (day 4), they would be associated with a positional index of 1, 3, and 4. For the static patient attributes, we encode them as shown in Fig. 3a and pad appropriately to produce a vector of same size as the $\lbrace h^{C}_i \rbrace$, and further combine with the positional offset of zero to produce ${\hat{X}}_0$.

4 $$\begin{aligned} H'&= f_{select}(GRU({\hat{X}}_0, {\hat{X}}_{1}, \ldots , {\hat{X}}_{T_h})), \quad f_{select} \text { returns the last element from GRU output sequence.} \end{aligned}$$

5 $$\begin{aligned} H'&= f_{select}(BERT({\hat{X}}_0, {\hat{X}}_{1}, \ldots , {\hat{X}}_{T_h})), \quad f_{select} \text { returns the first element from BERT output corresponding to ``CLS''.}\end{aligned}$$

6 $$\begin{aligned} H'&= FFN({\hat{X}}_0 \oplus {\hat{X}}_{1} \oplus \cdots \oplus {\hat{X}}_{T_h}), \text {where } \oplus \text { represents tensor concatenation operation}\end{aligned}$$

7 $$\begin{aligned} {\hat{o}}_t&= FFN(H') \end{aligned}$$

Finally, the resultant sequence from static patient attributes and the encoded vector embeddings from each time step (${\hat{X}}_0, {\hat{X}}_{1}, \ldots , {\hat{X}}_{T_h}$) are fed into a *temporal layer* (denoted as $f_T(\cdot$)). We experimented with $f_T(\cdot$) using the following: a GRU (Gated Recurrent Unit)^39^ layer, a feed forward network (FFN), and a multi-ahead attention layer^37^. The temporal layer is followed by a dense layer, and the output from the dense layer is passed through a non-linear function that produces the final scalar output. Handling of the input ($\lbrace {\hat{X}}_i \rbrace$) and the output from $f_T(\cdot$) changes depending on the specific choice of the temporal layer, and the specific implementations are described by equations (4)–(6). We use the binary cross-entropy as the loss function for training the temporal prediction layers combining $f_T(\cdot$) and the final dense layer.

## Results

### Comparison of SRD and COVID-19 Cohorts

Our motivation to adopt a transfer learning approach for training a COVID-19 outcome prediction model was inspired by the strong similarity between the vocabularies in SRD and COVID-19 cohorts (Table 1). 88.8% of the diagnosis codes in our COVID-19 cohort were also found in the SRD cohort. Similarly, the drugs, procedures and laboratory measurement codes in the COVID-19 cohort have an overlap of 94.04%, 63.2% and 90.57%, respectively. The SRD cohort also provided a strong coverage for patients with severe outcomes, even though the distributions of outcomes are slightly different from the COVID-19 cohort (Fisher’s exact test, $P < .05$). 14.6% of the patients in the SRD cohort required ventilation as compared to 11.4% in the COVID-19 cohort. Proportion of ICU days and mortality incidents in the SRD cohort are 23.4% and 8.34%, compared to 5.76% and 6.46% in the COVID-19 cohort, respectively.

**Table 1:** Summary of the COVID and SRD cohorts.

	COVID	SRD	*P*
Number of patients	1701	6892	
Number of hospitalizations	1701	9348	
**Quarter-wise distribution of hospitalization**			
Before 2020	0	8574	
2020-Q1	35	559	
2020-Q2	329	202	
2020-Q3	350	13	
2020-Q4	566	0	
2021-Q1	421	0	
Length of stay, median (Interquartile range)	4.8 (2.8–8.8)	5.0 (2.0–13.0)	.42
Age at encounter, mean (SD)	56.8 (18.6)	38.14 (30.9)	$<.001$
Age at encounter among adults, mean (SD)	56.8 (18.7)	60.11 (18.4)	$<.001$
**Age groups**			$<.001$
$<\,18$	0 (0%)	3689 (39%)	
18–30	166 (10%)	492 (5%)	
30–65	903 (53%)	2534 (27%)	
$$ >=$$65	632 (37%)	2633 (28%)	
**Race**			$<.001$
White	582 (34%)	3522 (51%)	
Black or African American	70 (4%)	314 (5%)	
Asian	228 (13%)	1157 (17%)	
American Indian or Alaskan Native	10 (1%)	21 (0%)	
Native Hawaiian or Other Pacific Islander	46 (3%)	173 (3%)	
Other/unknown	765 (45%)	1705 (25%)	
**Ethnicity**			$<.001$
Hispanic or Latino	691 (41%)	1687 (24%)	
Not Hispanic or Latino	989 (58%)	5099 (74%)	
Other/Unknown	21 (1%)	106 (2%)	
**Sex**			$<.001$
Male	854 (50%)	3108 (45%)	
Female	847 (50%)	3784 (55%)	
**Clinical outcomes**			
Ventilation (yes/no)	194/1507	1365/7983	$<.001$
ICU admissions (yes/no)	98/1603	2188/7160	$<.001$
Mortality (died/survived)	110/1591	780/8568	.01
**Input codes, [common codes/COVID total], [SRD total]**			
Diagnosis	2310/2599	6293	
Procedure	1204/1905	5778	
Drugs	2147/2283	4592	
Lab measurements	1355/1496	2431	

In terms of demographics, the COVID-19 cohort is quite different from the SRD cohort. In particular, the age distribution of patients in the two cohorts is significantly different (chi-squared $P < .001$, see Table 1). This is partly due to the fact that we restricted the COVID-19 cohort to adults only. However, if we restrict the analysis to adults only in both cohorts, the difference persists—while the proportion of patients in the age group 18–30 is quite similar (9.8% and 8.7% in COVID-19 and SRD cohorts, respectively), the COVID-19 cohort has a significantly higher proportion of patients in the 30–65 age group (53.1%) than the SRD cohort (44.8%) (chi-squared $P < .001$), and consequently, the mean age among adults in the COVID-19 cohort is significantly less than in the SRD cohort (Mann-Whitney U-statistic $P < .001$). The cohorts are significantly different with respect to sex (Fisher exact test $P < .001$), with the COVID-19 cohort being more balanced than the SRD cohort. There are significant differences between the cohorts with regard to race as well (chi-squared $P < .001$, ignoring the “Other/Unknown” class). Finally, the “Hispanic or Latino” ethnicity is significantly over-represented in the COVID-19 cohort compared to the SRD cohort (chi-squared $P < .001$).

### Training and evaluation setup

As mentioned earlier, we evaluate the model on two binary classification tasks on the COVID-19 dataset. To generate input dataset per patient, first, each patient’s stay duration is segmented into intervals of fixed length (*aggregation windows*) and the visit data within each interval is aggregated. For interpretability reasons, we use a 24-h aggregation interval. We then use the sliding window approach to generate individual samples for all the models by considering each timestep in the visit as the current timestep. No timesteps containing or following the first occurrence of a positive outcome can be part of an input. We benchmark our model against three methodologies: logistic regression (LR)^17^, a Gated Recurrent Unit (GRU)-based approach^18^, and BEHRT^32^ which is a state-of-the-art extension of BERT^37^ models for electronic healthcare records. Similar to BEHRT, MedBERT^33^ proposed training at patient visit sequence level, and uses only the diagnosis codes from a patient cohort. Hence, we only empirically compare with BEHRT which uses a much wider scale of structured EHR data and is more suitable for the in-stay patient study. We did not consider any time-series model due to the sparse and highly irregular nature of the time-series based laboratory measurements. The COVID-19 cohort was split randomly into 60% train, 20% validation, and 20% test dataset for evaluation purposes. The same split of patient cohorts was used for evaluating all of the methods. The models are evaluated using the AUROC (Area under Receiver Operating Characteristic curve) and the F1-score measure. The F1-score captures both the precision (positive predictive value) and recall (aka sensitivity, the fraction of relevant instances correctly retrieved) capability of the model. If TP, FP, and FN indicate “True Positive”, “False Positive” and “False Negative”, respectively, F1-score measure is given by:

$$\begin{aligned}&recall = \frac{TP}{TP+FN}, \quad precision = \frac{TP}{TP+FP}, \quad F1 = \frac{2 \times recall \times precision }{recall + precision} \end{aligned}$$

For each outcome task (the likelihood of the patient staying in the hospital and ventilation risk) we study two variations of prediction into the future: predict short-term (3 days) and long-term (7 days) ($T_f=3,7$) patient outcomes. For all variations, we feed 2 days of patient’s past history ($T_h$) to the model. A detailed performance analysis of ${\textsc {TransMED}}$ as a function of input history size and look-ahead duration is provided in Supplementary Table 4.

### Implementation details

#### Baselines

For LR, we use an aggregated view of patient’s diagnoses, procedures, drugs, lab codes, lab measurements, demographics, and risk factors over time. The model is trained using all variables encoded with the one-hot encoding scheme. BEHRT model adapts the BERT layers for structured EHR data. It considers the sets of medical codes occurring across multiple visits of a patient as a single instance of training sample. Analogous to the NLP domain, each code is embedded similar to a word, and a time-offset embedding is added for each token depending on the visit id. To ensure a fair comparison , the BEHRT model is pre-trained on SRD and fine-tuned on COVID-19 dataset as well. For the GRU baseline, we encode each input timestep as a multi-hot vector of diagnosis, procedure, and medication codes. Additional implementation details for the baseline methods can be found in Supplementary section titled “Methods: Implementation details.”

${{\varvec{{\textsc {TransMED}}}}}$: The BERT encoder layers in our model are implemented using the PyTorch BERT implementation available from Huggingface https://github.com/huggingface/transformers and used 2 layers and 2 heads with a hidden size of 64 for most configuration (refer to Supplementary section titled “Methods: Implementation details” for hyperparameter search). We noticeably used a low number of parameters in the model to ensure training convergence with limited data. We mask and predict one token for every patient state input to the model. The model was trained for a maximum of 300 epochs (both at visit level and patient temporal level) or if the validation loss stopped reducing for 15 consecutive epochs. Training was performed using a single NVIDIA Tesla V100 GPU of 16 GB memory capacity, leading to average training time of 2 h for the transfer learning step (using SRD cohort) and 30 min for the temporal modeling layer using COVID-19 cohort. For reproducibility purposes, our code will be made publicly available upon the acceptance of this paper.

### Performance analysis

We perform extensive experiments to answer following major questions: (1) which method is the best modeling approach for a specific prediction task? (2) What is the impact of using transfer learning for predicting COVID-19 patient outcomes? (3) How effective are different modalities of data in capturing patient state over various complexities of prediction objective?

Table 2 provides a comparison of ${\textsc {TransMED}}$ with respect to other benchmarks on all prediction tasks. We experimented with different combinations of input data sources for ${\textsc {TransMED}}$, LR, GRU, and BEHRT, and report the best performance for each model. All studies are performed in sliding window setting as described in the evaluation setup. A primary observation from Table 2 is that the performance gaps between the benchmarked methods vary depending on the prediction task. Arguably, the patient stay prediction is a simpler task since it requires developing a coarser-level understanding of patient’s severity. A patient can stay in the hospital for a variety of reasons and learning the association between all potential factors and a severity level is key to accurate prediction. On the other hand, predicting a patient’s ventilation risk requires reasoning about a more specific set of symptoms. Also, accurate prediction of ventilation risk requires robustness against label imbalance due to the rareness of the outcome. ${\textsc {TransMED}}$ performs on par with GRU for the patient stay prediction task and consistently outperforms logistic regression and BEHRT. For the two ventilation prediction tasks, ${\textsc {TransMED}}$ demonstrates an average improvement of 17.5% for AUROC and 34.84% for F1 measure over logistic regression, the next best performing method. A detailed comparison of the AUROC profiles for ${\textsc {TransMED}}$ and other baselines is provided in Supplementary Fig. 1.

**Table 2:** Performance comparison of ${\textsc {TransMED}}$ with other methods.

Model	3 days		7 days	
	AUROC	F1	AUROC	F1
**(a) Patient stay prediction results**				
LR	0.79 (0.77–0.81)	0.67 (0.65–0.71)	0.74 (0.71–0.77)	0.68 (0.65–0.72)
BEHRT	0.68 (0.64–0.71)	0.43 (0.42–0.44)	0.62 (0.58–0.65)	0.43 (0.42–0.44)
GRU	0.84 (0.81–0.86)	**0.77 (0.75–0.79)**	0.80 (0.76–0.83)	0.67 (0.61–0.72)
TransMED	**0.84 (0.82–0.86)**	0.72 (0.70–0.74)	**0.80 (0.77–0.83)**	**0.73 (0.70–0.76)**
**(b) Ventilation risk prediction results**				
LR	0.64 (0.52–0.78)	0.31 (0.28–0.35)	0.68 (0.56–0.80)	0.31 (0.28–0.34)
BEHRT	0.63 (0.60–0.66)	0.43 (0.42–0.44)	0.66 (0.63–0.69)	0.2 (0.19–0.22)
GRU	0.62 (0.48–0.77)	0.5 (0.5–0.5)	0.72 (0.59–0.87)	0.51 (0.49–0.54)
TransMED	**0.83 (0.77–0.89)**	**0.52 (0.49–0.56)**	**0.77 (0.67–0.87)**	**0.53 (0.49–0.57)**

Significant values are in bold.

The methods are evaluated for predicting patient stay and ventilation risk in short-term (next 3 days) as well as long-term (next 7 days). ${\textsc {TransMED}}$’s best performance was observed using BERT as fine tuning layer for long term ventilation prediction while feed-forward layer did best for other tasks.

#### Impact of transfer learning and hierarchical model

 With regards to self-supervised learning approaches, $${\textsc {TransMED}}$$ demonstrates an average gain of 25.6% for AUROC and 45.8% for F1 measures over BEHRT across four tasks. The significant out-performance of $${\textsc {TransMED}}$$ over BEHRT demonstrates the impact of our hierarchical modeling approach beyond adapting BERT and pre-training on multiple cohorts. The impact of transfer learning and multi-modality is studied in Table 3. $${\textsc {TransMED}}$$$$_{w/o\ TL}$$ lists performance without transfer learning from SRD (pretrains and finetunes only on COVID-19 cohort) and compares it to different pretraining settings. Transfer learning makes a significant impact in improving performance across all four tasks, with an average improvement of 12.9% and 10.3% in AUROC for patient stay and ventilation prediction, respectively. Noticeably, transfer learning is the primary contributing factor in the significant performance difference between $${\textsc {TransMED}}$$ and other baseline methods for the ventilation prediction tasks. This demonstrates transfer learning helps learn the fine grained interactions between medical concepts that are essential to accurate prediction of complex medical outcomes, in presence of limited training data. For multi-modality, we see that, using only the procedure codes can offer significant predictive performance for all methods for the patient stay prediction task (see Supplementary Table 5). Introduction of risk factors from clinical notes, laboratory measurements and demographics however improves the accuracy of ventilation prediction by 3.7% and 4.0% in AUROC, respectively. The combination of demographics, laboratory measurements, clinical notes and procedure codes consistently produces the best performance for $${\textsc {TransMED}}$$ across four prediction settings. Supplementary Table 5 provides the details for all input variations that were experimented with and reports the performance breakdown for $${\textsc {TransMED}}$$ as a function of input features.

**Table 3:** Ablation study results of the proposed ${\textsc {TransMED}}$ model analyzing the impact of transfer learning and data modalities on the final performance.

Method	Short-term patient stay		Long-term patient stay		Short-term ventilation		Long-term ventilation	
	AUROC	F1	AUROC	F1	AUROC	F1	AUROC	F1
**Impact of transfer learning**								
$${{\varvec{{\textsc {TransMED}}}}}$$$$_{w/o\ TL}$$	0.71	0.62	0.72	0.67	0.72	0.47	0.71	0.48
$${{\varvec{{\textsc {TransMED}}}}}$$$$_{with\ SRD}$$	0.81	0.69	0.78	0.70	0.79	0.50	0.74	0.49
$${{\varvec{{\textsc {TransMED}}}}}$$$$_{with\ SRD+COVID}$$	0.83	0.71	0.79	0.72	0.84	0.52	0.77	0.51
**Impact of data modalities**								
$${{\varvec{{\textsc {TransMED}}}}}$$$$_{struct}$$	0.82	0.70	0.76	0.69	0.81	0.54	0.74	0.50
$${{\varvec{{\textsc {TransMED}}}}}$$$$_{struct+NLP}$$	0.83	0.71	0.77	0.69	0.79	0.53	0.75	0.51
$${{\varvec{{\textsc {TransMED}}}}}$$$$_{struct+NLP+demo}$$	0.83	0.72	0.77	0.70	0.81	0.53	0.75	0.51
$${{\varvec{{\textsc {TransMED}}}}}$$$$_{struct+NLP+demo+labvalues}$$	0.83	0.71	0.79	0.72	0.84	0.52	0.77	0.51

Ablation study was performed with a fixed set of hyper-parameters and feed forward network fine tuning layer. See Supplementary “Methods: Implementation details” for description of hyperparameter tuning.

### Model interpretability via single and multicomorbidity analysis

This section presents two different evaluations of our model’s ability to account for important clinical factors. First, we begin with profiling the model’s predicted risk score distribution in terms of well-established univariate risk factors in the clinical literature^29,30^, namely the chronic conditions extracted from clinical notes and demographics information such as age, gender, and sex. Next, we examine the ability of the model to account for important multi-comorbidities. Taking inspiration from the literature on recommendation systems^40^, we demonstrate a new methodology to identify top multi-comorbidities present in the patient population. We then compute a ranked list of the top multi-comorbidities associated with patients with a ventilation outcome, and compare the top-ranked multi-comorbidities as predicted by the model. This is a significantly harder test and we demonstrate that ${\textsc {TransMED}}$ successfully ranks the majority of the multi-comorbidities using this top-k verification approach.

#### Defining clinical risk factors

**Figure 4:**
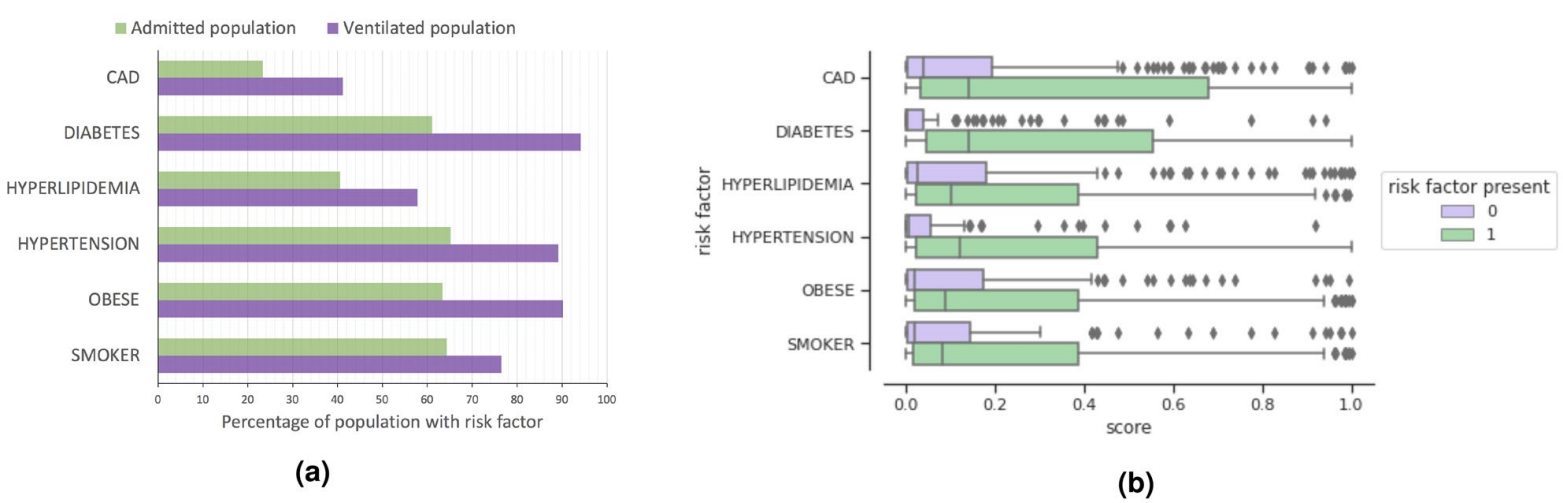
(**a**) Risk factor prevalence in ventilated patients compared to the total population. (**b**) The distribution of model predicted risk scores for ventilation outcomes across the test cohort. The bars show the range while the mean score is showed as a line across the bar. All chronic conditions lead to a higher predicted risk while the mean scores were highest for patients with CAD and Diabetes [consistent with the ground truth trends observed for ventilated patients in (**a**)].

Figure 4a shows the distribution of different chronic conditions and risk factors in the entire population vs ventilated population. We observe that the admitted patients had high prevalence of hypertension, obesity, smoking, and diabetes (present in around 60-65% of the population), while hyperlipidemia and coronary artery disease (CAD) were observed in 40% and 23% of the cohort, respectively. However, the prevalence of all risk factors was substantially higher in the ventilated population. Diabetes, obesity, and hypertension were present in more than 90% of patients, while CAD and diabetes patients had the highest ratio of (ventilated/total population).

#### Univariate analysis of model predicted risk scores

Figure 4b provides the model predicted ventilation risk score for patients with or without a risk factor. Patients with any of the risk factors were predicted to have a higher risk than patients without a risk factor, which is consistent with ground truth observation. The average predicted risk scores were highest for CAD and diabetes patients, with CAD patients having the largest variance. Between all risk factors, diabetes and hypertension patients had the highest increase in risk compared to the non-diabetic or non-hypertension patients.

**Figure 5:**
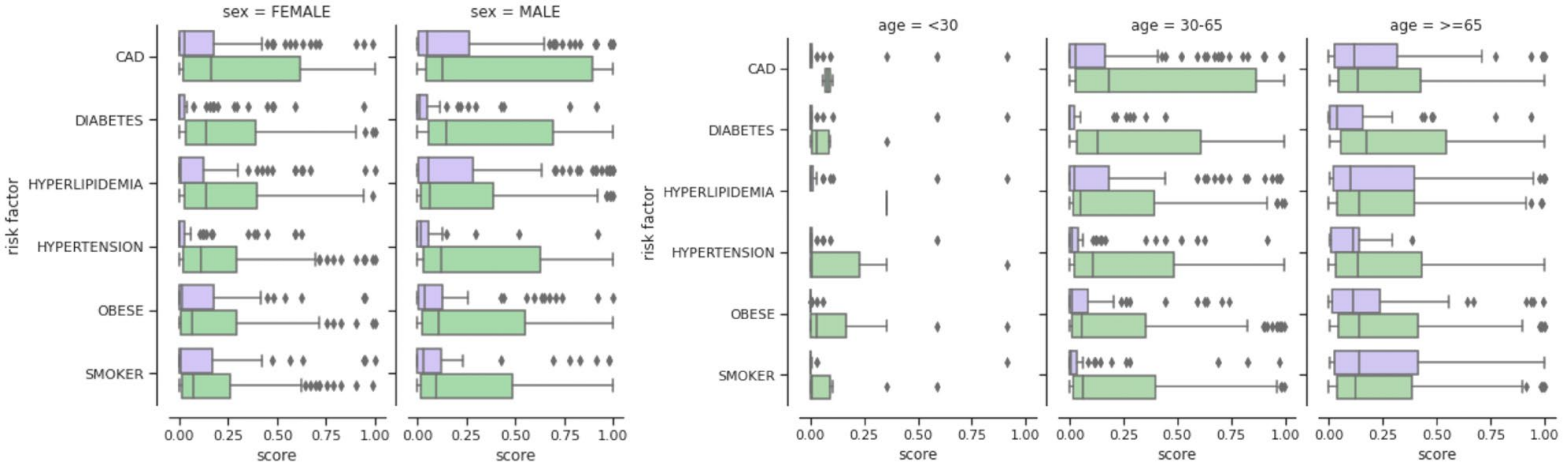
The influence of chronic risk factors across sex and age groups on the ${\textsc {TransMED}}$ predicted risk scores for ventilation outcome ($T_f=3$).

We further studied the model predicted risk scores across different patient demographics in terms of age and sex (shown in Fig. 5). Patient race was excluded from our analysis due to insufficient coverage across different racial groups. Amongst male and female sex, the model predicted higher risk scores for male patients compared to their female counterparts, consistent with the ground truth for ventilation cases observed at 7.9% in male and 4.3% in female patients, respectively. For different age groups, the patients under 30 years of age were predicted to be at very low risk even in the presence of risk factors, again consistent with ventilation outcomes observed in the data (1/164 patients under 30). The model predicted risk increased for patients with age when they did not have any risk factor. However, in the presence of a chronic condition, the model gave a larger range of risk scores to CAD patients between ages 30–65, although the mean scores for other chronic conditions remained similar across ages 30-65 (ventilated = 51/883) and the 65+ age group (ventilated = 50/614). A detailed analysis of model performance for these sub-cohorts via AUROC measures is provided in Supplementary Table 2 and 3.

#### Multivariate analysis of model predicted risk scores

**Figure 6:**
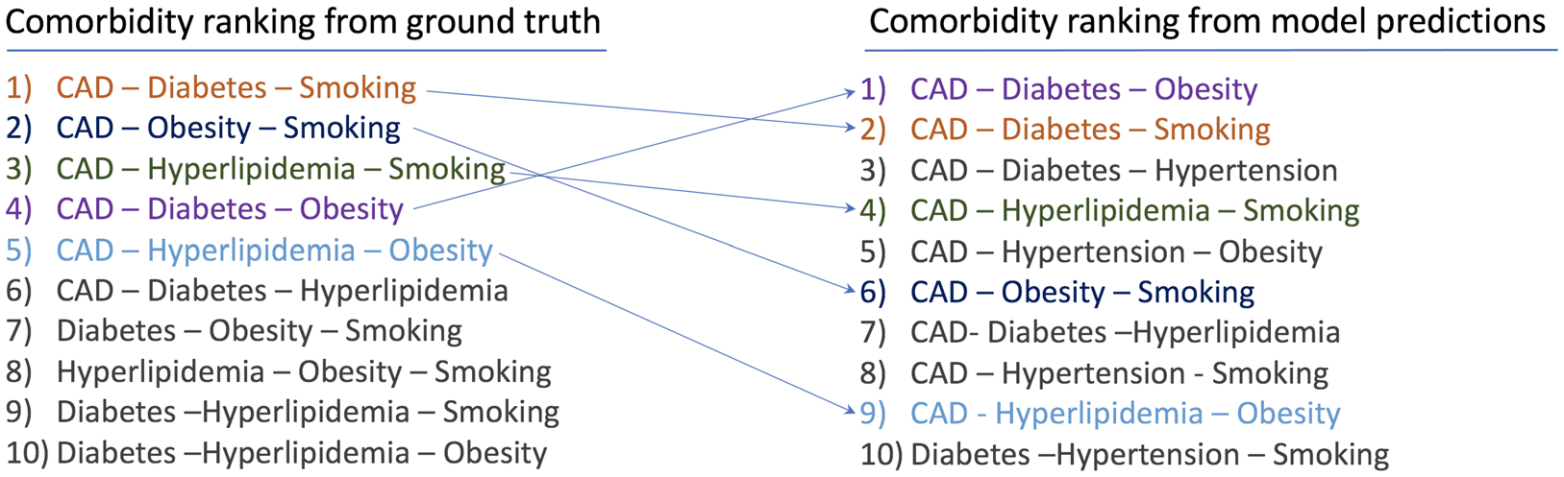
Size-3 comorbidities by prevalence in ventilated patients vs model predicted risk score. The top 5 (out of 30) comorbidities in the ground truth were found within the top 9 risk scores predicted by the model.

Finally, we introduce our methodology for evaluating the model predictions in terms of multi-way feature interactions. Details of the multi-comorbidity generation and ranking process are provided in Supplementary “Implementation of Multi-Comorbidity Ranking” section. We compare a ranking of the top multi-comorbidities from six clinical risk factors as determined by their prevalence in the ventilated sub-cohort with a ranking derived through model predicted scores (Fig. 6). The model predicted risk ranking on the right closely agrees with the ground truth ranking of comorbidities for ventilated patients, with top-5 (out of 30) ground truth comorbidity interactions, in top-9 comorbidities identified by the model. Our analysis establishes that the ${\textsc {TransMED}}$ learns reliable risk scores across salient clinical risk factors and captures multi-way feature interactions consistent with ground truth observed for ventilation outcomes.

## Discussion

Set against the backdrop of COVID-19, we reviewed a number of challenges faced by health systems worldwide to develop improved risk stratification tools for pandemic responses. We created a rigorous model evaluation framework dedicating significant effort to explore the best settings for the benchmark methods, exploring different pre-training strategies (pre-train on only SRD cohort, or a merged SRD and COVID-19 patient cohort) and evaluating impact of data source selection (see results for 12 different combinations in Supplementary Table 5. The evaluations were performed across the four prediction task settings of varying complexity. Our conclusions are drawn from the resultant space of models representing the widest possible variation of data and neural architectures for our study cohorts. The following are key observations that emerge from our study.

### Transfer learning from existing disease can be key to modeling emerging infectious (and rare) diseases

 Pre-training ${\textsc {TransMED}}$’s transformer-based multi-modal patient encoder layers on the larger SRD cohort consistently provided performance boost ranging from 8 to 17% for our model performance. While our insight into the experiments with transfer learning primarily arose from clinical intuitions, we confirmed the “transfer potential” by inspecting the overlap in the vocabularies, as well as similarities in outcomes as detailed in the previous section. To our knowledge, no other work has demonstrated the ability to train an effective deep learning model for COVID-19 by training on other pre-existing patient cohorts.

### Building models with imperfect multi-modal data

Much of the initial literature on predictive models for electronic healthcare records is overwhelmingly biased towards models with diagnostic codes and drug codes. Studies based on procedure codes or time-series measurements constitute a small fraction. ${\textsc {TransMED}}$’s best performance was driven by a combination of procedure codes, numeric laboratory measurements, demographics and knowledge of risk factors extracted from clinical notes. Given the historical importance of diagnosis codes and drug codes in building predictive models and their relative under-performance in our study raises key questions about the utility of each data source. As Fig. 1 illustrates, we found diagnostic codes are coded in sparsely. While medication data is available more continuously, it does not change frequently to suggest changes in a patient’s condition. From this perspective, our best performing input combination is strongly intuitive. Observing critical procedures such as a radiological test or heparin therapy allows a model to escalate a patient’s severity level, observing measurements such lymphocyte counts over time allows the model to reason about the trend of infection levels, and the knowledge of demographic information such as age, race, and sex coupled with prior knowledge of baseline risk factors such as diabetes, hypertension, and CAD can guide a model’s association with other symptoms and outcomes.

### Guiding training data complexity 

We observe that in a setting with imperfect data, we need to explicitly reason about the discriminative value of each data source. We also ensured the availability of sufficient training data for each feature introduced in every data source. We only included 8 laboratory measurements out of 1496 unique laboratory measurement codes by considering the number of patients who had available data (we set 1200 patients as a minimum threshold) and the minimum number of days results were available for each patient (set to two). Ensuring high overlap between medical codes was critical to the benefit of transfer learning as well. Considering that procedure codes have an overlap of 63.2% across SRD and COVID-19 patient cohorts, as compared to 88.88% for diagnostic codes, 94.04% for drugs, and 90.57% for laboratory measurements, it is safe to say that the discriminative nature of procedure codes was a dominant factor over vocabulary overlap. Introduction of each feature affects the learning complexity by increasing the number of model parameters. Implicitly and intuitively, we sought to maximize the ratio of information entropy in our training data for each model parameter.

### Merit of hierarchical approach for learning from sparse data

We conclude this discussion by noting that our approach outperformed others by explicitly recognizing the sparsity in the training data. Instead of learning the association of different medical codes or laboratory measurements at an entire patient stay level, we sought to learn the dependencies at finer granularity of time. However, we also used the demographics information and risk factors as static attributes associated with each time interval. Given that most of the multi-modal data streams occur sparsely and irregularly, this design decision reduced the complexity of learning the association between “everything” but provided less ambiguous input for each sample in the transfer learning step. In short, we ensured that the information flowed across modalities within a single time interval via the pre-training/transfer learning step, and then across time through the temporal layer during the fine-tuning step. Given that both ${\textsc {TransMED}}$ and BEHRT were trained on identical transfer learning settings, the strong performance gap of ${\textsc {TransMED}}$ over BEHRT demonstrates the merit of our hierarchical modeling approach.

## Summary and conclusions

Our work shows that a transfer-learning approach that learns from prior and related EHR databases is a promising way to build predictive models for diseases with limited data. A key conclusion from our study is that hierarchical learning, that first models the interaction between various medical concepts over shorter intervals, and then learns temporal dependencies is effective for transfer learning across diseases where patient conditions evolve at different time-scales. Our methodology demonstrates that a neural architecture that integrates both static (such as demographics and clinical risk factors) and dynamic information (such as temporal lab measurements) in a fashion that is robust to the sparsity and irregularity of multi-modal data sources is likely to provide the best predictive model for complex outcomes. We also propose a method for multi-way comorbidity analysis that can be extended to include a richer set of phenotypes and evaluate a model’s ability to capture complex interactions between them. ${\textsc {TransMED}}$’s ability to improve the prediction accuracy on complex tasks such as predicting the likelihood of ventilation seven days into the future by an average of 17.5% on AUROC and 34.84% for F1 score demonstrates the promise of our method and motivates further investigation for tasks such as mortality prediction and drug recommendation.

## Supplementary Information

Supplementary Information.

## Data Availability

The data that support the findings of this study are available from STAnford medicine Research data Repository (STARR) (https://starr.stanford.edu) but restrictions apply to the availability of these data, which were used under license for the current study, and so are not publicly available. The code will be released as open source on https://github.com/pnnl/TransMED.
