# Supplementary material for: Preparing for the next pandemic via transfer learning from existing diseases with hierarchical multi-modal BERT: a study on COVID-19 outcome prediction
Source: Sci Rep. 2022 Jun 24;12:10748. doi: 10.1038/s41598-022-13072-w (PMC9232529; doi:10.1038/s41598-022-13072-w)
Supplement: Supplementary file 1 — Supplementary Information. [file 41598_2022_13072_MOESM1_ESM.pdf]

Supplementary Information for

Preparing for the next pandemic via transfer learning from existing diseases with hierarchical multi-modal BERT: a study on COVID-19 outcome prediction

Khushbu Agarwal[1], Sutanay Choudhury*[1], Sindhu Tipirneni[2], Pritam Mukherjee[3], Colby Ham[1], Suzanne Tamang[4], Matthew Baker[6], Siyi Tang[5], Veysel Kocaman[7], Olivier Gevaert[3,4], Robert Rallo[1], and Chandan K Reddy[2]

[1]Pacific Northwest National Laboratory, Richland, 99354, USA
[2]Department of Computer Science, Virginia Tech, Arlington, 22203, USA
[3]Stanford Center for Biomedical Informatics Research, Department of Medicine, School of Medicine, Stanford University, Stanford, 94305, USA
[4]Department of Biomedical Data Science, Stanford University, Stanford, 94305, USA
[5]Department of Electrical Engineering, Stanford University, Stanford, 94305, USA
[6]Division of Immunology and Rheumatology, Department of Medicine, Stanford University, Stanford, 94305, USA
[7]John Snow Labs, Delaware City, 19958, USA
*Sutanay.Choudhury@pnnl.gov

Data Sources

Entity Extraction from EHRs with Assertion and Patient Attribution

Extracting valuable information from EHRs with intelligent systems starts with Named entity recognition (NER), a key building block of common NLP tasks such as question answering, topic modelling, information retrieval, etc[1]. In the medical domain, NER plays the most crucial role by giving out the first meaningful chunks of a clinical note, and then feeding them as an input to the subsequent downstream tasks such as clinical assertion status[2], clinical entity resolvers [3] and de-identification of the sensitive data[4]. However, segmentation of clinical and drug entities is considered to be a difficult task in biomedical NER systems because of complex orthographic structures of named entities [5].

As the risk factor extraction from clinical notes would play a pivotal role in representing the patient context, doing this automatically, accurately, and at scale is highly desired. In order to ensure this, we decided to deploy Spark NLP for Healthcare library[6] that has state-of-the-art NER models pretrained on curated datasets as well as well known benchmark datasets[2,7,8]. NER implementation in Spark NLP is based on the BiLSTM-CNN-Char framework, a modified version of the architecture proposed by Chiu et.al.[9]. It is a neural network architecture that automatically detects word and character-level features using a hybrid bidirectional LSTM and CNN architecture, eliminating the need for most feature engineering steps.

In addition to extracting clinical entities from the text, we also constrained the assertion status for the extracted to be “present". This ensures that a patient is not associated with diabetes by processing a sentence such as “patient does not have diabetes." Similarly, we also enforce dependency checks to ensure a condition is associated with the patient and not someone else. This would avoid associating the patient with diabetes from a sentence such as “father has diabetes." The deep neural network architecture for assertion status detection in Spark NLP is based on a Bi-LSTM framework, and is a modified version of the architecture proposed by Fancellu et.al.[10]. Spark NLP has its own NER model to extract the patient risk factors such as coronary artery disease (CAD), diabetes, hyperlipidemia, hypertension, history of taking long-term medications, obesity, smoking. Named as ner risk factors, it is trained on the dataset shared during 2014 i2b2/UTHealth shared task Track 2 [11]. The assertion model that is fed by the out of NER model is also an out of the box solution that is pretrained on a combination of a dataset shared at 2010 i2b2/VA challenge[2].

Selection of Severe Respiratory Disease Cohort

The severe respiratory disease cohort patients are defined by having an inpatient visit with ARDS or pneumonia or influenza diagnosis and cross-referenced with procedural codes for ventilatory support. The ICD-9 used for specification of each of the diagnosis and ventilatory support are provided in Table 1.

ICD9	Description of diagnostic codes
518.82	Other pulmonary insufficiency, not elsewhere classified
518.x5	Pulmonary insufficiency following trauma and surgery
96.70	Continuous invasive mechanical ventilation of unspecified duration
96.71	Continuous invasive mechanical ventilation for less than 96 consecutive hours
96.72	Continuous invasive mechanical ventilation for 96 consecutive hours or more
480.x	Pneumococcal pneumonia [Streptococcus pneumoniae pneumonia
481.x	Other bacterial pneumonia
482.x	Other bacterial pneumonia
483.x	Pneumonia due to other specified organism
484.x	Pneumonia in infectious diseases classified elsewhere
485.x	Bronchopneumonia, organism unspecified
486.x	Pneumonia, organism unspecified
487.x	Influenza
488.x	Influenza due to certain identified influenza viruses

Supplementary Table 1. Description of Diagnosis Codes for identifying patients with Ventilatory Support Codes (96.*), ARDS (518.*), Influenza and Pneumonia (PHX) (48*.*)

Methods: Implementation details

Logistic regression: All observations of a patient are aggregated across the input observation time for input to the logistic regression (LR) model. The lab measurements across the observation period are averaged for feeding into the model. Specifically, we created a binary feature for each code in the dataset, created one binary/categorical/continuous feature as appropriate for each of the demographic variables, created one binary feature for each risk factor, and one continuous feature for each of the laboratory measurements. We trained LR using Adam optimizer with a learning rate of 5×10^{-4} and a batch size of 256. The training is stopped when the validation F1 score does not improve for 40 epochs.

BEHRT: The BEHRT model is pre-trained on SRD by predicting masked codes in the input (representing all of the patient data observed over the course of time). The model is then fine-tuned on COVID-19 dataset by adding a pooling and sigmoid layer as suggested in the model architecture [12]. For BEHRT, the embedding dimension is set to 64, the number of Transformer layers to 4, and number of attention heads in each layer to 4. For training, we use Adam optimizer with a learning rate of 5×10^{-4} and a batch size of 64. The training is stopped when the validation F1 score does not improve for 10 epochs.

GRU: Each input vector is passed through a dense layer with 32 units before passing it to a GRU cell with 64 hidden units. The last hidden state is then transformed by a dense layer with softmax to output the two class probabilities. For training, we use Adam optimizer with a learning rate of 5×10^{-4} and a batch size of 64. The training is stopped when the validation F1 score does not improve for 10 epochs.

All the baselines have been implemented in Tensorflow. For each baseline, we experimented with different subsets of modalities and showed the result for the best case. For patient stay prediction with lookahead 3, LR and GRU showed best

Supplementary Table 2. Model performance (AUROC) across age and sex subcohorts for patient stay prediction

		Patient Stay - next 3 days	Patient Stay - next 7 days
**sex**			
	FEMALE	0.813 (0.781 - 0.840)	0.778 (0.724 - 0.825)
	MALE	0.832 (0.803 - 0.859)	0.783 (0.743 - 0.822)
**age (yrs)**			
	< 30	0.843 (0.763 - 0.923)	0.719 (0.477 - 0.931)
	30 - 65	0.866 (0.842 - 0.888)	0.826 (0.790 - 0.861)
	≥ 65	0.759 (0.719 - 0.794)	0.719 (0.658 - 0.768)

performance using only procedure codes, and BEHRT showed best performance using measurement codes. For patient stay prediction with lookahead 7, LR showed best performance using only procedure codes, GRU showed best performance using only diagnosis, medication, and procedure codes, and BEHRT showed best performance using all codes. For ventilation task with lookahead 3, LR showed best performance using only diagnosis codes, GRU showed best performance using only diagnosis and procedure codes, and BEHRT showed best performance using only measurement codes. For patient stay prediction with lookahead 7, LR showed best performance using only all codes and lab values, GRU showed best performance using only diagnosis, medication, and procedure codes, and BEHRT showed best performance using only measurement codes.

TRANSMED: We experimented with ADAM and stochastic gradient descent (SGD) optimizers and varying learning rates [0.1, 0.01, 0.001, 0.0001]. SGD with learning rate of 0.1 was associated with best results. The ReduceLROnPlateau scheduler was used for dynamic learning rate reduction via validation experiments. We also experimented with number of BERT layers and number of attention heads [2-12] each, as suggested in the original implementation [13]. Two layers and two heads performed best for all configurations, except for long term ventilation prediction task where best performance was achieved using 12 attention heads.

Implementation of Multi-Comborbidity Ranking

To compute the ranking based on prevalence, we iterated over each patient to generate all possible 3-tuples (such as [“CAD", “diabetes", “smoking"]) from the associated risk factors. Next, we ranked the generated sets based on their counts in the ventilated patient population.

To compute a multi-comorbidity ranking based on model predictions, we first created patient subgroups where each subgroup corresponds to a multi-comorbidity as specified above. Note that the subgroups can be overlapping since a patient associated with (“CAD", “diabetes", “smoking") can also be part of another subgroup corresponding to (“CAD", “diabetes", “obesity"), if the patient had four of these risk factors. Next, we used the predicted ventilation risk score for each patient in a subgroup and averaged to generate a subgroup level score. Finally, we ranked the multi-comorbidities based on the averaged predicted ventilation risk score.

Additional Results

Performance across demographics based sub-cohorts: We further characterize the model accuracy across different sex and age groups(Table 2, 3) across the 4 different prediction task settings. We observed minimal difference in accuracy of patient stay across male and female patients. However, we observe higher model accuracy for ventilation of male over female population. This is largely due to the fewer number of female patients with a ventilation outcome (7.9% in male and 4.3% female) in the available COVID-19 cohort. Across the age groups, the accuracy is again largely observed varying as a function of number of positive training instances available for each cohort. The patient stay was more accurately predicted for age group 30 to 65, while ventilation was more accurately predicted for patients equal to or more than 65 years old.
TRANSMED temporal performance: In addition, to the existing tasks for long term and short term prediction for patient stay and ventilation, we perform a thorough evaluation of TRANSMED's temporal prediction performance. We study the prediction accuracy of our system as function of number of days of observation (input time steps T_h) and number of look-ahead days into the future at the end of observation period T_f. Table (4a) and (4b) shows the performance of model for patient stay and ventilation tasks. As expected, the prediction accuracy of the model decreases as we try to predict farther into the future. Interestingly, first 2 days of input time steps were the most effective setting for predicting across all outcomes due to insufficient number of data samples (average length of stay for both cohorts was less than 5 days, Table 1 in the primary manuscript).

Supplementary Table 3. Model performance (AUROC) across age and sex subcohorts for predicting ventilation

		Ventilation - next 3 days	Ventilation - next 7 days
**sex**			
	FEMALE	0.792 (0.722 - 0.873)	0.598 (0.266 - 0.848)
	MALE	0.857 (0.778 - 0.942)	0.775 (0.706 - 0.874)
**age (yrs)**			
	< 30	-	-
	30 - 65	0.789 (0.680 - 0.889)	0.601 (0.356 - 0.860)
	≥ 65	0.866 (0.779 - 0.961)	0.781 (0.714 - 0.873)

Supplementary Table 4. Model performance (AUROC) as a function of history length T_h and look-ahead time T_f. The experiments were performed using a fixed set of hyper-parameters for the model, with 2 BERT encoder layers, 2 attention heads and a learning rate of 0.01 with SGD optimizer.

(a) Predicting patient stay

**Input window size**	**Lookahead window**		
	**2 days**	**3 days**	**7 days**
**2 days**	**0.857 (0.834 - 0.878)**	**0.808 (0.784 - 0.830)**	**0.783 (0.747 - 0.816)**
**4 days**	0.847 (0.817 - 0.874)	0.790 (0.757 - 0.823)	0.748 (0.697 - 0.800)
**8 days**	0.805 (0.760 - 0.849)	0.759 (0.707 - 0.813)	0.715 (0.651 - 0.780)

(b) Prediction of ventilation

**Input window size**	**Lookahead window**		
	**2 days**	**3 days**	**7 days**
**2 days**	**0.863 (0.807 - 0.919)**	**0.855 (0.797 - 0.913)**	**0.809 (0.751 - 0.884)**
**4 days**	0.835 (0.742 - 0.926)	0.795 (0.710 - 0.888)	0.748 (0.672 - 0.843)
**8 days**	0.704 (0.591 - 0.799)	0.765 (0.689 - 0.898)	0.553 (0.468 - 0.638)

AUROC results: The detailed plots for area under receiver operating characteristics for each of the 4 tasks across all methods are shown in Figure 1. As discussed earlier, TRANSMED outperforms on both ventilation tasks while GRU and TRANSMED coincide closely for patient stay prediction. A threshold of 0.5 was used for the classification performance evaluation.

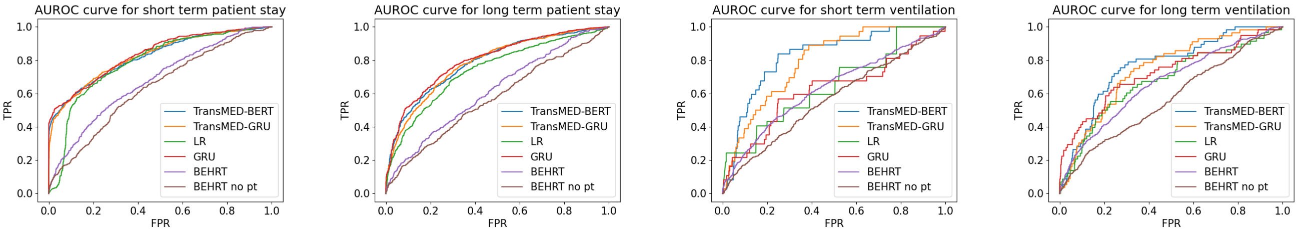

(a) (b) (c) (d)

Supplementary Figure 1. AUROC curves comparing all benchmarks and TRANSMED ablation study performance across the four prediction tasks with varying lookahead distances. (a) patient stay (3 days), (b) patient stay (7 days), (c) ventilation (3 days), (d) ventilation (7 days).

Supplementary Table 5. Model performance (AUROC) with various combinations of input sources: diagnostic codes (Dx), drug codes (Rx), procedure codes (Proc), laboratory measurements (Meas), and clinical notes (Notes).

Model Inputs	Short-term patient stay		Long-term patient stay		Short-term ventilation		Long-term ventilation	
	AUROC	F1	AUROC	F1	AUROC	F1	AUROC	F1
Dx	0.52	0.51	0.53	0.34	0.49	0.49	0.52	0.50
Rx	0.49	0.49	0.49	0.34	0.53	0.50	0.43	0.49
Meas	0.51	0.43	0.52	0.34	0.43	0.50	0.57	0.50
Proc	0.82	**0.71**	0.77	0.68	**0.82**	**0.53**	0.74	0.47
Dx + Rx	0.49	0.52	0.52	0.34	0.52	0.50	0.50	0.05
Notes + Proc	**0.83**	**0.71**	**0.78**	**0.71**	**0.82**	0.51	0.73	0.47
Dx + Proc + Rx	0.51	0.49	0.50	0.34	0.51	0.50	0.52	0.49
Demo + Notes + Proc	**0.83**	**0.71**	**0.78**	0.70	0.81	**0.53**	**0.77**	**0.52**
Dx + Meas + Proc + Rx	0.51	0.38	0.50	0.35	0.54	0.50	0.49	0.41
Demo + Dx + Meas + Proc + Rx	0.58	0.52	0.60	0.59	0.54	0.43	0.48	0.40
Demo + Meas_val + Notes + Proc	0.82	**0.71**	**0.78**	**0.71**	0.80	0.51	0.76	0.50
Demo + Dx + Meas + Notes + Proc + Rx	0.69	0.62	0.70	0.65	0.60	0.40	0.59	0.39

Related Work

The disruptive impact of deep learning in natural language processing inspired extension of prominent NLP methods into EHR prediction tasks. Treating codes such as diagnostic codes, drugs and procedures as sets of words led to adoption of convolutional neural networks[14], sequence modeling approaches such as skip-gram models and variants of recurrent neural networks[15–21] and transformers[12,22–25] for various EHR prediction tasks.

Our neural architecture is distinct from the other recent BERT or Transformer-based architectures such as BEHRT[12] and MedBERT [25] models in multiple dimensions. Similar to BEHRT, MedBERT proposed training at patient visit sequence level, and uses only the diagnosis codes from a patient cohort. Hence, we only empirically compare with BEHRT which uses a much wider scale of structured EHR data and is more suitable for the in-stay patient study. The encoder layer in our model integrates temporal information in multiple representations, that includes discrete entity-based representation of diagnostic codes, drug codes, procedure codes along with continuous valued time-series measurement of laboratory tests. To the best of our knowledge, the proposed model demonstrates the widest integration of EHR-based data sources across multiple modalities for COVID-19 severity prediction.

Learning representations of medical codes has been an area for active research by itself. We learn contextual representations of each medical code such that the embedding for a diagnosis code such as “diabetes" can differ from patient to patient, or even for the same patient, it’s vector embedding can change depending on other codes it co-occurs with. Medical codes such as diagnosis codes and drug codes are naturally organized in hierarchical ontologies[26]. While we do not exploit such structure in our work, we foresee room for further performance improvement by leveraging on prior literature on learning hierarchical representations of the codes[22,27–29] as well as the relationships between diagnosis codes and drug codes[18].

Two particular threads are relevant to our work in this broad landscape: 1) approaches for integrating diverse data sources and modalities, and 2) leveraging on prior data sources. Fewer efforts have focused on integrating multiple data modalities in EHR databases. Inclusion of static attributes of patients such as race, sex, ethnicity, and other patient health risk factors have been shown to improve model performance when incorporated into LSTM and CNN models[30]. Integration of clinical notes into a multi-modal model has been done via extraction of UMLS concept identifiers (CUIs)[31] and emotional states[32]. RAIM[20] uses lab measurements and interventions to guide attention weights over time-series of high-density waveforms and vital signs.[21] propose an LSTM-based time-aware multi-modal encoder that processes time-series, diagnoses, demographics, and medications for identifying sepsis subphenotypes. However, we did not consider any time-series model due to the sparse and highly irregular nature of thetime-series based laboratory measurements.Last but not the least, pre-training with larger datasets that may be similar, transfer learning has been shown to improve model performance on small or limited healthcare datasets[33,34].

References in Supplementary Information

1. Yadav, V. & Bethard, S. A survey on recent advances in named entity recognition from deep learning models. arXiv preprint arXiv:1910.11470 (2019).
2. Uzuner, Ö., South, B. R., Shen, S. & DuVall, S. L. 2010 i2b2/va challenge on concepts, assertions, and relations in clinical text. J. Am. Med. Informatics Assoc. **18**, 552–556 (2011).

3. Tzitzivacos, D. International classification of diseases 10th edition (icd-10):: main article. CME: Your SA J. CPD **25**, 8–10 (2007).
4. Uzuner, Ö., Luo, Y. & Szolovits, P. Evaluating the state-of-the-art in automatic de-identification. J. Am. Med. Informatics Assoc. **14**, 550–563 (2007).
5. Liu, S., Tang, B., Chen, Q. & Wang, X. Effects of semantic features on machine learning-based drug name recognition systems: word embeddings vs. manually constructed dictionaries. Information **6**, 848–865 (2015).
6. Kocaman, V. & Talby, D. Spark nlp: Natural language understanding at scale. Softw. Impacts **8**, 100058 (2021).
7. Yang, X. et al. A study of deep learning methods for de-identification of clinical notes in cross-institute settings. BMC Med. Informatics Decis. Mak. **19**, 232 (2019).
8. Henry, S., Buchan, K., Filannino, M., Stubbs, A. & Uzuner, O. 2018 n2c2 shared task on adverse drug events and medication extraction in electronic health records. J. Am. Med. Informatics Assoc. **27**, 3–12 (2020).
9. Chiu, J. P. & Nichols, E. Named entity recognition with bidirectional lstm-cnns. Transactions Assoc. for Comput. Linguist. **4**, 357–370 (2016).
10. Fancellu, F., Lopez, A. & Webber, B. Neural networks for negation scope detection. In Proceedings of the 54th annual meeting of the Association for Computational Linguistics (volume 1: long papers), 495–504 (2016).
11. Stubbs, A., Kotfila, C., Xu, H. & Uzuner, Ö. Identifying risk factors for heart disease over time: Overview of 2014 i2b2/uthealth shared task track 2. J. biomedical informatics **58**, S67–S77 (2015).
12. Li, Y. et al. Behrt: transformer for electronic health records. Sci. reports **10**, 1–12 (2020).
13. Devlin, J., Chang, M.-W., Lee, K. & Toutanova, K. Bert: Pre-training of deep bidirectional transformers for language understanding. arXiv preprint arXiv:1810.04805 (2018).
14. Nguyen, P., Tran, T., Wickramasinghe, N. & Venkatesh, S. Deepr: a convolutional net for medical records. IEEE journal biomedical health informatics **21**, 22–30 (2016).
15. Choi, E. et al. Retain: An interpretable predictive model for healthcare using reverse time attention mechanism. In Advances in Neural Information Processing Systems, 3504–3512 (2016).
16. Baytas, I. M. et al. Patient subtyping via time-aware lstm networks. In Proceedings of the 23rd ACM SIGKDD international conference on knowledge discovery and data mining, 65–74 (ACM, 2017).
17. Ma, F. et al. Dipole: Diagnosis prediction in healthcare via attention-based bidirectional recurrent neural networks. In Proceedings of the 23rd ACM SIGKDD international conference on knowledge discovery and data mining, 1903–1911 (ACM, 2017).
18. Choi, E., Xiao, C., Stewart, W. F. & Sun, J. Mime: Multilevel medical embedding of electronic health records for predictive healthcare. arXiv preprint arXiv:1810.09593 (2018).
19. Zhang, J., Kowsari, K., Harrison, J. H., Lobo, J. M. & Barnes, L. E. Patient2vec: A personalized interpretable deep representation of the longitudinal electronic health record. IEEE Access **6**, 65333–65346 (2018).
20. Xu, Y., Biswal, S., Deshpande, S. R., Maher, K. O. & Sun, J. Raim: Recurrent attentive and intensive model of multimodal patient monitoring data. In Proceedings of the 24th ACM SIGKDD International Conference on Knowledge Discovery & Data Mining, 2565–2573 (ACM, 2018).
21. Yin, C., Liu, R., Zhang, D. & Zhang, P. Identifying sepsis subphenotypes via time-aware multi-modal auto-encoder. In Proceedings of the 26th ACM SIGKDD international conference on knowledge discovery & data mining, 862–872 (2020).
22. Shang, J., Ma, T., Xiao, C. & Sun, J. Pre-training of graph augmented transformers for medication recommendation (2019). 1906.00346.
23. Zhang, X. et al. Inprem: An interpretable and trustworthy predictive model for healthcare. In Proceedings of the 26th ACM SIGKDD International Conference on Knowledge Discovery & Data Mining, 450–460 (2020).

24. Luo, J., Ye, M., Xiao, C. & Ma, F. Hitanet: Hierarchical time-aware attention networks for risk prediction on electronic health records. In Proceedings of the 26th ACM SIGKDD International Conference on Knowledge Discovery & Data Mining, 647–656 (2020).

25. Rasmy, L., Xiang, Y., Xie, Z., Tao, C. & Zhi, D. Med-bert: pretrained contextualized embeddings on large-scale structured electronic health records for disease prediction. NPJ digital medicine **4**, 1–13 (2021).

26. Agarwal, K. et al. Snomed2vec: Random walk and poincar\'e embeddings of a clinical knowledge base for healthcare analytics. arXiv preprint arXiv:1907.08650 (2019).

27. Choi, E., Bahadori, M. T., Song, L., Stewart, W. F. & Sun, J. Gram: graph-based attention model for healthcare representation learning. In Proceedings of the 23rd ACM SIGKDD international conference on knowledge discovery and data mining, 787–795 (2017).

28. Zhang, M., King, C. R., Avidan, M. & Chen, Y. Hierarchical attention propagation for healthcare representation learning. In Proceedings of the 26th ACM SIGKDD International Conference on Knowledge Discovery & Data Mining, 249–256 (2020).

29. Lu, C., Reddy, C. K. & Ning, Y. Self-supervised graph learning with hyperbolic embedding for temporal health event prediction (2021). 2106.04751.

30. Suresh, H. et al. Clinical intervention prediction and understanding using deep networks (2017). 1705.08498.

31. Chowdhury, S., Zhang, C., Yu, P. S. & Luo, Y. Mixed pooling multi-view attention autoencoder for representation learning in healthcare (2019). 1910.06456.

32. Ji, S. et al. Suicidal ideation detection: A review of machine learning methods and applications. IEEE Transactions on Comput. Soc. Syst. **8**, 214–226, DOI: 10.1109/TCSS.2020.3021467 (2021).

33. Gupta, P., Malhotra, P., Vig, L. & Shroff, G. Transfer learning for clinical time series analysis using recurrent neural networks (2018). 1807.01705.

34. Zhang, X. S., Tang, F., Dodge, H., Zhou, J. & Wang, F. Metapred: Meta-learning for clinical risk prediction with limited patient electronic health records (2019). 1905.03218.
